# P02.11. Adherence and satisfaction with the experimental mind and body intervention in the LIFE weight loss maintenance study

**DOI:** 10.1186/1472-6882-12-S1-P67

**Published:** 2012-06-12

**Authors:** C Elder, L DeBar, K Funk, W Vollmer, N Lindberg, C Ritenbaugh, G Meltesen, C Gallison, V Stevens

**Affiliations:** 1 Kaiser Permanente Center for Health Research, Portland, USA; 2 University of Arizona, Tucson, USA; 3 Healing Touch Acupuncture, Portland, USA

## Purpose

To provide an in depth analysis of adherence and satisfaction with the mind and body intervention for participants in the LIFE weight loss maintenance trial.

## Methods

We performed a secondary analysis of 142 obese participants who had lost at least 10 pounds in a conventional weight loss program and who were randomized to the experimental weight loss maintenance intervention. This experimental intervention consisted of instruction and application of an energy psychology intervention, Tapas Acupressure Technique (TAT®). TAT practice combined self-acupressure with a prescribed set of mental steps. Participants were advised to practice TAT at home daily. The main outcome measure was self reported frequency of TAT practice.

## Results

Sixty-six percent of TAT participants attended at least six of the eight intervention sessions, and the drop out rate was 3.5%. Almost half of TAT participants reporting practicing TAT at home on average for 2-3 days per week, while 8% reported on average zero days per week of TAT practice and 2% reported daily TAT practice. Sixty-two percent reported practicing less than 10 minutes/session, while 27% reported practicing 10-20 minutes/session. Satisfaction was significantly correlated with less weight regain (p=.001), and a majority of participants reported that they were at least somewhat likely to continue using TAT. Frequency of TAT home practice was not significantly associated with changes in weight, stress, insomnia, depression, or quality of life.

## Conclusion

The data suggest moderate acceptance and adherence with the TAT intervention. However our analyses showed no association between frequency of TAT home practice and clinical outcomes. Further research is required toward identifying and achieving optimal levels of home practice in clinical trials of energy psychology techniques.

